# DGD-CNet: Denoising Gated Recurrent Unit with a Dropout-Based CSI Network for IRS-Aided Massive MIMO Systems

**DOI:** 10.3390/s24185977

**Published:** 2024-09-14

**Authors:** Amina Abdelmaksoud, Bassant Abdelhamid, Hesham Elbadawy, Hadia El Hennawy, Sherif Eldyasti

**Affiliations:** 1 Electronics and Communications Department, Faculty of Engineering, Modern Academy for Engineering and Technology, Cairo 11585, Egypt; 2 Electronics and Communications Department, Faculty of Engineering, Ain Shams University, Cairo 11517, Egypt; bassant.abdelhamid@eng.asu.edu.eg (B.A.); hadia.elhennawy@gmail.com (H.E.H.); 3 Network Planning Department, National Telecommunication Institute, Cairo 11768, Egypt; heshamelbadawy@ieee.org; 4 Electronics and Communications Department, Arab Academy for Science, Technology and Maritime Transport, Cairo 11799, Egypt; Sherifkh@aast.edu

**Keywords:** channel estimation, CSI feedback, deep learning, Denoising Gated Recurrent Unit, DGD-CNet, dropout technique, FDD, IRS, Massive MIMO, NMSE, system accuracy

## Abstract

For the deployment of Sixth Generation (6G) networks, integrating Massive Multiple-Input Multiple-Output (Massive MIMO) systems with Intelligent Reflecting Surfaces (IRS) is highly recommended due to its significant benefits in reducing communication losses for Non-Line-of-Sight (NLoS) conditions. However, the use of passive IRS presents challenges in channel estimation, mainly due to the significant feedback overhead required in Frequency Division Duplex (FDD)-based Massive MIMO systems. To address these challenges, this paper introduces a novel Denoising Gated Recurrent Unit with a Dropout-based Channel state information Network (DGD-CNet). The proposed DGD-CNet model is specifically designed for FDD-based IRS-aided Massive MIMO systems, aiming to reduce the feedback overhead while improving the channel estimation accuracy. By leveraging the Dropout (DO) technique with the Gated Recurrent Unit (GRU), the DGD-CNet model enhances the channel estimation accuracy and effectively captures both spatial structures and time correlation in time-varying channels. The results show that the proposed DGD-CNet model outperformed existing models in the literature, achieving at least a 26% improvement in Normalized Mean Square Error (NMSE), a 2% increase in correlation coefficient, and a 4% in system accuracy under Low-Compression Ratio (Low-CR) in indoor situations. Additionally, the proposed model demonstrates effectiveness across different CRs and in outdoor scenarios.

## 1. Introduction

Recently, the use of efficient modern technologies in wireless communication systems, including Massive Multiple-Input Multiple-Output (Massive MIMO), in addition to Intelligent Reflecting Surface (IRS) has gained significant attention. This is because these technologies have succeeded in improving the spectrum utilization of communication systems, making them more efficient [1]. Despite the high cost and energy consumption, which are inherent in wireless communication systems, several wireless communication protocols have integrated Massive MIMO systems, to improve their Spectral Efficiency (SE), as well as their Energy Efficiency (EE) [2]. This feature enhances the connectivity of a transmitter/receiver by facilitating more reliable connections and a higher data transmission rate. One of the major obstacles facing Massive MIMO technology is channel estimation [3]. This is because of substantial performance degradation, where many operations, such as beamforming and resource allocation, require precise Channel State Information (CSI). Additionally, in the Frequency Division Duplex (FDD)-based Massive MIMO, obtaining precise downlink CSI presents a significant challenge because of the excessive overhead needed for a feedback link to the Base Station (BS), which represents an important step in knowing the downlink channel characteristics [4].

To further decrease communication losses resulting from Non-Line-of-Sight (NLoS) and increase SE/EF of Massive MIMO systems, they have been recently integrated with IRS. The latter has attracted significant interest as a potential technology, due to its ability to establish a communication channel between User Equipment (UE) and BS in the presence of towering structures or other obstacles [5]. Many reflecting elements, characterized by passivity and low cost, constitute the IRS system [6]. The passive IRS reflects the incident signal with an appropriate phase shift, despite its inability to perform signal processing and amplification [7]. This technological advancement has the potential to enhance network coverage, lessen interference, and enhance the accuracy of communication systems [8]. However, due to the inability of numerous passive IRS components to transmit and receive data, estimating the channel in wireless communication aided by the passive IRS is difficult. Given the fact that the BS-IRS and IRS-UE channels cannot be individually estimated, an estimated cascaded BS-IRS-UE channel is obtained. However, cascaded channels face two significant challenges in terms of channel estimation, namely accuracy limitation and huge training overhead [9].

The literature has mostly focused on the estimation of CSI under the Time Division Duplex (TDD) system-based Deep Learning (DL) techniques. This can be seen in [10,11,12,13,14]. These studies employ DL-based techniques in their models, leveraging the assumption that channels are reciprocal since the BS can obtain the uplink CSI to estimate the downlink CSI. On the other hand, IRS-aided Massive MIMO systems, under the FDD system, are limited, despite FDD being an essential operating system for applications requiring low latency and high reliability. Since the FDD system has no channel reciprocity, the UE must relay the downlink CSI to BS via the feedback link. This introduces challenges in FDD-based IRS-aided Massive MIMO systems due to the increased overhead and the potential for inaccurate channel estimation of the cascaded CSI. Compressive Sensing (CS) techniques have been employed to reduce the feedback overhead. However, they pose challenges such as noise sensitivity and sub-optimal performance in time-varying and complex environments. Therefore, recent research is directed on leveraging DL-based models in the context of FDD channel estimation to enhance the accuracy and efficiency of CSI estimation. Despite these advancements, adapting these techniques to dynamic channel conditions and balancing the trade-offs between estimation accuracy and feedback overhead remain ongoing challenges.

Advanced DL-based models, including the Gated Recurrent Unit (GRU) and Dropout (DO) technique, have been developed to improve the accuracy and efficiency of CSI estimation. GRUs [15,16] use built-in memory cells to store information over time, whereas the DO [17,18] increases model robustness by deactivating neurons at random during training to prevent overfitting.

Building on these advancements, this paper proposes a DL-based model in the feedback link of FDD-based IRS-aided Massive MIMO, with the following essential contributions:

- Proposing a regulation technique called “Dropout (DO)” within the framework of our proposed model in [19] named “Channel state information Network, in conjunction with the Denoising Convolution Neural Network (CsiNet-DeCNN)”. This addition will mitigate overfitting, which can take place during the learning process and will take into consideration its effects on system performance since reducing the training overhead is accompanied by an improvement in system accuracy.
- Leveraging a Recurrent Neural Network (RNN), known as the Gated Recurrent Unit (GRU), in our proposed model enhances the ability to discriminate and preserve essential signal features throughout the denoising process. The GRU improves the channel estimation accuracy and facilitates the learning of spatial structures in conjunction with time correlation in time-varying channels.

So that the validity of the proposed contribution may be assisted throughout this paper by investigating the performance of the proposed DGD-CNet model which undergoes a comprehensive analysis of different parameters, these parameters may be summarized as follows: Normalized Mean Square Error (NMSE), correlation coefficient, the system accuracy, the Signal-to-Noise-Ratio, as well as the computational complexity. The obtained results may be consolidated to prove that the proposed model can have higher system performance. This may be achieved by reducing the training overhead and achieving more accurate channel estimation at BS.

The rest of this paper is divided into the following: Section 2 presents previous work undertaken in the same field, within literature. Section 3 presents an IRS-aided Massive MIMO communication system model and the CSI feedback process. Section 4 describes the proposed model to be used in channel estimation. Section 5 discusses the results and analysis. Finally, a conclusion of the research and recommendations for future work can be found in Section 6.

## 2. Related Work

To address the challenge of channel estimation, researchers have proposed various techniques in the literature for its enhancement in Massive MIMO [20] and IRS-aided Massive MIMO systems [21]. The authors in [22,23] introduced models based on CS, which decrease CSI feedback overhead and improve accuracy in FDD-based Massive MIMO systems. In [22], a CS model reduced feedback overhead by using the spatial representation of signals, which improved efficiency in managing CSI. However, this model may face challenges in noisy or rapidly changing environments, where the assumption of spatial sparsity may not accurately capture real-world complexities, while [23] utilized CS techniques to estimate CSI in slow-varying channels, which helped in reducing feedback overhead and improving efficiency; this model has limitations in fully addressing system complexity and enhancing accuracy in more dynamic or noisy environments.

Recently developed DL-based models possess a notable benefit when applied to the FDD-based Massive MIMO CSI estimation and feedback link. This is due to their ability to autonomously obtain the characteristics of a given problem and eliminate the requirements for prior extensive knowledge. In [24], researchers proposed a model, which was explored for the first time. This included the use of a DL-based model for feedback that utilized CsiNet, employing the channel matrix in Massive MIMO. This channel matrix functions as an image. To compress and receive feedback of the CSI, a Convolutional Neural Network (CNN) is used. CsiNet is composed of a number of Neural Network (NN) layers [25], that are designed to minimize CSI feedback while recovering the channel with high accuracy. NN comprises an input layer, several hidden layers, and output layers. It is responsible for training and learning the model, allowing it to generate accurate predictions.

Due to concerns related to network training, inaccurate channel estimation, and ignorance of the time correlation of the channel, the adoption of RNN has increased in recent years. A key feature of RNN is the presence of a hidden layer capable of retaining previously processed information. During the processing of time series data, this layer represents a structural advantage. Consequently, RNN works as a channel estimator, to improve the accuracy and learning of the CSI estimation. To estimate the channel for the feedback link, the authors in [26,27] integrated the CsiNet used in [24] with a Long Short-Term Memory (LSTM) network, i.e., an RNN, which utilizes time-variant parameters. In [26], the authors proposed a wireless channel temporal and frequency correlation-based CsiNet-LSTM, which learns spatial structure. This system is coupled with time correlation. For the latter, samples used to train time-varying Massive MIMO channels were employed. In [27], a CsiNet architecture integrated a Convolutional (Conv) layer with an LSTM-based compression and decompression model (Conv-LSTMCsiNet), to improve CSI prediction and independently extract the spatial and temporal features. Although both models presented in [26,27] achieved better results than CsiNet, limitations arose due to the complexity of LSTMs, with their multiple gates and separate memory cells, leading to an increase in the space complexity of the model. The proposed model, however, uses GRU to mitigate these issues.

In [28], a two-stage model was introduced, where the first stage utilized uplink channel estimation employing an Adaptive Deep Neural Network (ADNN), optimizing channel amplitude estimation and reconstruction. The second stage involved hybrid precoding for downlink data transmission using Adaptive LSTM (ALSTM) with performance improved by optimizing the hidden neurons. While this approach enhances accuracy in both channel estimation and data recovery, it also increases the risk of overfitting. As a result, the model performs well in controlled environments requiring high precision but may struggle in more dynamic environments due to these limitations. In [29], the authors proposed an information detection and selection network (IdasNet), a DL-based framework for compressing CSI and providing transmitter feedback in an FDD-based Massive MIMO system. However, its complex design may complicate implementation in real-world applications. Moreover, its performance can be affected if the pre-compression and self-information selection steps are not optimally executed.

To enhance both the accuracy and efficiency within the communication network, IRS was used. Researchers in [11,12,13,14] introduced an enhanced channel estimation model, to be used with the TDD-based IRS-aided Massive MIMO system. In [11], an Improved Deep Residual Shrinkage Network (IDRSN) was introduced to improve the pilot design by effectively reducing noise, making it advantageous in stable channel conditions. However, it may perform worse in dynamic environments due to its potential difficulty in adapting to sudden changes, leading to less accurate channel estimation. The authors in [12] used a Residual U-shaped Network (ResU-Net) and a deep CS-based channel estimation model to identify the cascaded channel matrix with minimal pilot overhead. However, this model may struggle in scenarios with rapidly changing channels, potentially impacting the channel estimation accuracy. In [13], researchers presented a hybrid IRS structure and DL-based CNN for sparse channel amplitude determination. However, the added complexity of the model may decrease channel estimation accuracy, making it less suitable for real-time applications. In [14], to denoise channel estimation, a Convolutional Deep Residual Network (CDRN) was used in IRS-Multi-User Communication systems (MUCs). However, its performance may degrade in highly dynamic channels.

Currently, limited research is being conducted on DL-based CSI feedback for FDD-based IRS-aided Massive MIMO systems. Our proposed model in [19] focuses on a DL technique to determine accurate CSI in FDD-based IRS-aided Massive MIMO systems, specifically through the feedback link. This model works on reducing channel noise through the integration of CsiNet and a deep denoising convolution neural network (CsiNet-DeCNN). The DeCNN exploits spatial characteristics of noisy channel matrices and subtracts noise additively, improving estimation accuracy. The results obtained through CsiNet-DeCNN were better than those achieved by the CsiNet model [24] in channel reconstruction. However, it does not address the time correlation in time-varying channels, which is crucial for real-life applications. In [30], the author introduced an attention mechanism-based CsiNet (ACNet), which uses a limited number of parameters, with the consideration of time correlation in time-varying channels. Although this model outperforms other DL-based CsiNet models in the literature, its performance decreases with a decreased Compression Ratio (CR), defined as the ratio of the size of the compressed data to the size of the original data.

Hence, this paper proposed a model that focuses on enhancing channel estimation for the feedback link in FDD-based IRS-aided Massive MIMO systems. This incorporates considerations for time correlation in time-varying channels, resulting in improved performance even with decreased CRs. Table 1 summarizes the aforementioned works relating to channel estimation models and the proposed model.

Notation: Throughout this paper, scalar variables, vectors, and matrices are represented by normal-face letters, bold-face lowercase, and uppercase symbols, respectively. C represents the complex field, while R is the real field. The real and imaginary parts of a matrix **A** are represented by Re(**A**) and Im(**A**), respectively. The subscript ${\left(.\right)}^{H}$ denotes the Hermitian (or conjugate transpose) of a matrix or vector. The notation E{.} represents the expectation operation.

Additionally, the complex space of m × n dimensional matrices are represented by $C^{m\times n}$. The function diag(.) denotes the diagonal matrix formed from the input vector. The Euclidean norm is returned by the operator ${\left\|.\right\|}_{2}$, the Frobenius norm is denoted by ${\left\|.\right\|}_{F}$, the notation ${\left(.\right)}^{T}$ denotes the transpose of the matrix, and the operation ° is the Hadamard product.

## 3. System Model and CSI Feedback Process

A brief introduction of the FDD-based IRS-aided Massive MIMO communication system model will be presented in the following subsections, along with the inclusion of the CSI feedback process.

### 3.1. System Model

**Figure 1:**
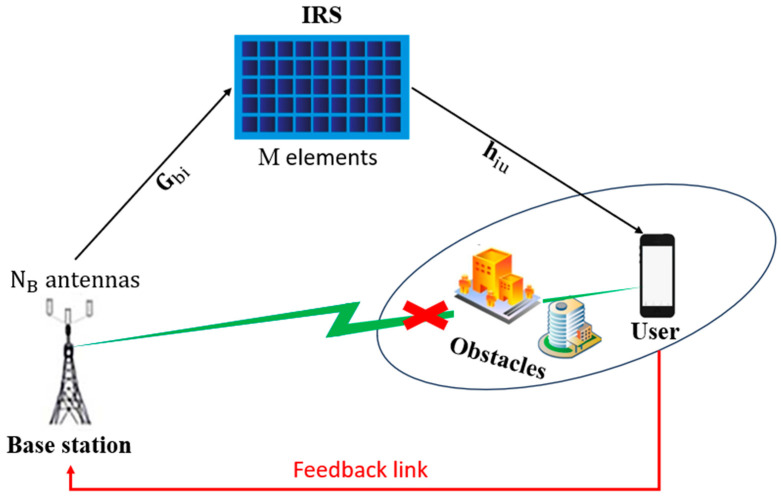
IRS-aided Massive MIMO system.

A downlink FDD-based Massive MIMO system with a single-antenna UE and $N_{B}$ antennas at the BS is proposed. Within this system, the estimation of the downlink CSI is made at the level of the UE. A feedback loop then goes back to the BS through the feedback link, as shown in Figure 1. Assuming that the UE is located within the dead zone service region, an IRS composed of M-reflecting elements is implemented in this scenario, to establish a communication channel between BS and UE. Let φ be the diagonal reflection coefficient matrix of the IRS. As per [19], it is represented as follows:

(1) $$\varphi =diag[{\beta_{1}e}^{j\theta_{1}},\beta_{2}e^{j\theta_{2}},\ldots \ldots \ldots ,{\beta_{M}e}^{j\theta_{M}}]\in C^{M\times M}$$

where $0\leq \beta_{m}\leq 1$ is the amplitude and $0\leq \theta_{m}\leq 2\pi$ is the phase shift of the m-th IRS element m=1,……, M,. The IRS elements are assumed to be a perfect reflecting surface, meaning that $\beta_{1}\;$= … =$\;\beta_{M}$ = 1, which has been assumed in several studies, such as [31,32].

The Orthogonal Frequency Division Multiplexing (OFDM) is used to transmit the signal with ${\tilde{N}}_{c}$ sub-carriers. Data symbols are pre-encoded by the BS and transmitted through the wireless channel in the downlink of the FDD system.

UE mobility leads to a time-varying channel. As such, the UE receives the following signal at time t on the n˗th sub-carrier [32]:

(2) $$y_{n,t}=h_{iu(n,t)}^{H}\varphi {G_{bi(n,t)}z}_{n,t}a_{n,t}+w_{n,t}$$

where $G_{bi(n,t)}\in C^{{M\times N}_{B}}$ is the BS-IRS channel and $h_{iu(n,t)}\in C^{M\times 1}$ is the IRS-UE channel at n-th sub-carriers, the precoding vector is $z_{n,t}\in C^{N_{B}\times 1}$, the data transmission symbol through BS is denoted by $a_{n,t}\in C$, and noise is represented by $w_{n,t}\in C$, which follows an Additive White Gaussian Noise (AWGN) distribution with zero mean and unit variance.

The cascaded effective channel vector $h_{n,t}\in C^{N_{B}\times 1}$ between BS and UE can be defined as in [32]:

(3) $$h_{n,t}^{H}=h_{iu(n,t)}^{H}\varphi G_{bi(n,t)}$$

### 3.2. CSI Feedback Process

**Figure 2:**
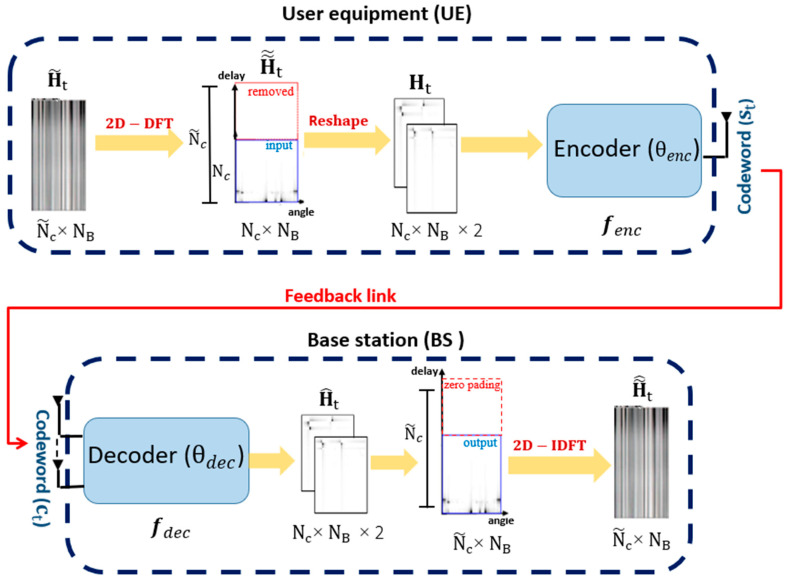
DL-based CSI encoder–decoder framework and the CSI feedback process.

Let ${{\tilde{H}}_{t}=[h_{1,t},h_{2,t},\ldots ,h_{{\tilde{N}}_{c},t}]}^{H}\in C^{{\tilde{N}}_{c}\times N_{B}}$ be the cascaded CSI, in the spatial domain for all sub-carriers at time t, as depicted in Figure 2.

The UE estimates and feeds ${\tilde{H}}_{t}$ back to the BS, in the FDD system, using the feedback link. All feedback parameters in the spatial domain are the size of ${\tilde{H}}_{t}$, as ${\tilde{N}}_{c}\times N_{B}$ is excessively large to be fed back over the restricted bandwidth, especially in Massive MIMO. This results in substantial feedback overhead. The channel matrix ${\tilde{H}}_{t}$ is transmitted to the angular-delay domain using a two dimensional-Discrete Fourier Transform (2D-DFT). This transformation helps reduce the feedback overhead [24,27]. This 2D-DFT is defined as follows [26]:

(4) $${\tilde{\tilde{H}}}_{t}=\;F_{N_{c}}{\tilde{H}}_{t}F_{N_{B}}^{T}$$

where $F_{N_{c}}$ and $F_{N_{B}}$ are ${\tilde{N}}_{c}\times {\tilde{N}}_{c}$ and $N_{B}\times N_{B}$ DFT matrices, respectively. Since multipath arrivals occur in a narrow period in the angular-delay domain, a small number of first ${\tilde{\tilde{H}}}_{t}$ elements contain a significant component. As for other components, they have more proximity to zero. Therefore, the initial $N_{c}$ rows of ${\tilde{N}}_{c}$ can be preserved, while the remaining rows are eliminated, and the resulting channel coefficient matrix dimensions are reduced from$\;{\tilde{N}}_{c}$ × $N_{B}\;$to $N_{c}\;$×$\;N_{B}$ in the angular-delay domain.

Since NN is incapable of handling complex numbers, the resulting channel coefficient matrix is partitioned into the real and the imaginary, with the values normalized to fall within the interval [0,1], to reach two channel coefficient matrices of $H_{t}$ with dimensions $N_{c}\times N_{B}$.

The BS receives a consistent and instantaneous estimated CSI from the UE. This is performed for the tracking of the time-varying channel. Therefore, the location of the UE fluctuates in real-time scenarios, resulting in doppler spread, while the surrounding environment remains relatively unchanged. To calculate the coherence time of the doppler spread, the following Equation is used [26]:

(5) $$T_{c}=\frac{c}{2vf_{o}}$$

where v is the UE movement velocity and $f_{o}$ represents carrier frequency; as for c, it marks the speed of light. Rather than individually reconstructing CSI to complete the step of reconstruction, the BS can contain not just the feedback, but also previous channel information. This includes a sequence of channel matrices ${\left\{H_{t}\right\}}_{t=1}^{T}=\{H_{1},\;H_{2},\ldots \ldots ,H_{T}\}$ in the angular-delay domain, where T represents the adjacent instantaneous angular-delay domain channel matrices, with the feedback time interval set to $\delta_{t}$. The group demonstrates the property of correlation, and T fulfills $0\leq \delta_{t}T\leq T_{c}$.

At the level of the UE, the encoder is employed to compress the CSI matrices of size ${L=2N}_{c}N_{B}$ into a codeword ($s_{t}$) of a K-dimensional vector, using a denoising encoder for compression, where the CR can be represented by

(6) $$CR=\frac{K}{L}$$

and the codeword can be encoded as

(7) $$s_{t}=f_{enc}(Re\left(H_{t}\right),Im\left(H_{t}\right),\theta_{enc})$$

where $f_{enc}$ (.) and $\theta_{enc}$ indicate the compression function and the parameter of the encoder, respectively. Particularly, $f_{enc}$ includes a series of Conv Layers, denoising processes, activation functions, and dimensionality reduction. Comprehensive details regarding the architecture of the encoder and processing steps will be provided in Section 4. The encoder compresses the CSI matrices to a K-dimensional vector $s_{t}$ ∈ $R^{K\times 1}$, with $K<<N_{c}\times N_{B}$. The BS then receives the codeword ($s_{t}$) in feedback, and the received codeword $c_{t}$ ∈ $R^{K\times 1}$ at BS can be denoted by

(8) $$c_{t}=s_{t}+n_{t}$$

where $n_{t}$ represents the AWGN, having a zero mean and unit variance.

At the level of the BS, the decoder network recovers the CSI matrices at time t, using the denoising and memory modules, which can extract the time correlation from the previously reconstructed channel matrices ${\hat{H}}_{1}$,……, ${\hat{H}}_{t-1}$. For the purpose of construction at this specific time, it adds these matrices to the received $c_{t}$. The reconstructed channel coefficient matrix of CSI with the dimensions $N_{c}\times N_{B}\times 2$ at time t is represented by

(9) $${\hat{H}}_{t}=f_{dec}(c_{t},\theta_{dec};{\hat{H}}_{1},\ldots \ldots ,\;{\hat{H}}_{t-1})$$

where $f_{dec}(.)$ is the decompression function of the decoder, $\theta_{dec}$ denotes decoder parameters. Then, the output CSI matrix is changed back to the spatial domain by padding zero rows and performing a 2D-Inverse DFT (2D-IDFT), to reach$\;{\hat{\tilde{H}}}_{t}$ with the dimensions of ${\tilde{N}}_{c}\times N_{B}$.

## 4. Proposed DGD-CNet Channel Estimation Model

This paper aimed to improve channel estimation accuracy by introducing the DO technique and GRU unit to the CsiNet-DeCNN model. Therefore, the following sub-sections provide a brief illustration of the CsiNet-DeCNN model, GRU, and DO, as well as the proposed DGD-CNet model architecture and the key performance indexes used to evaluate the proposed model.

### 4.1. CsiNet-DeCNN Model

**Figure 3:**
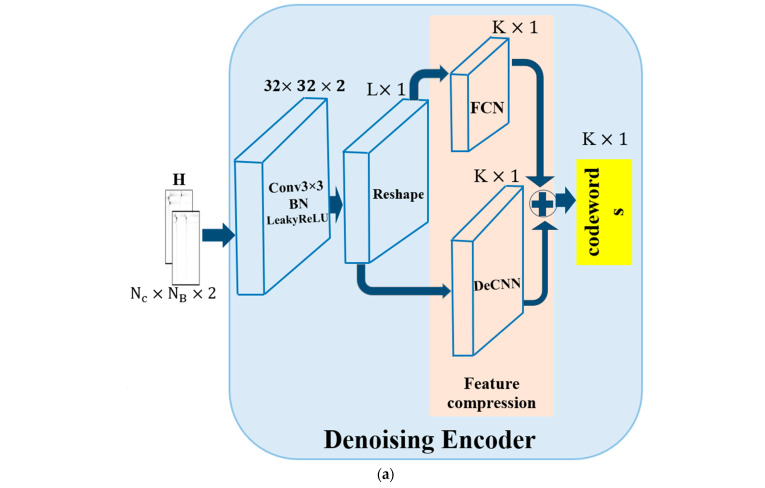

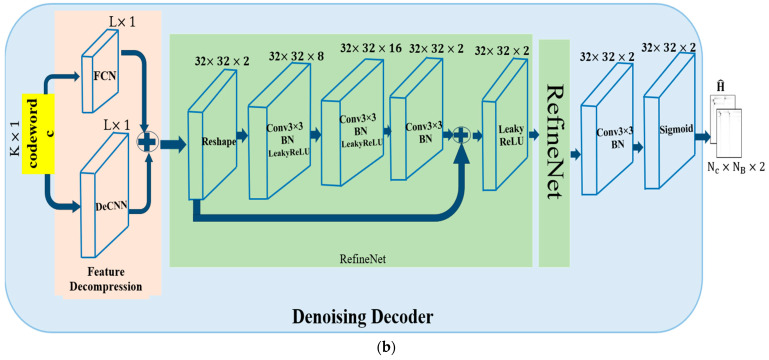
CsiNet-DeCNN model with a (**a**) Denoising Encoder and (**b**) Denoising Decoder.

Previously, our CsiNet-DeCNN model was published in [19], in which the denoising encoder–decoder model was proposed. It showed the effects of integrating the denoising module into the autoencoder CsiNet model. During the process of CSI sensing and reconstruction, CsiNet-DeCNN performed exceptionally well. As shown in Figure 3, the UE employs the denoising encoder for compressing the channel matrix H with $N_{c}\times N_{B}\times$2.

The Leaky Rectified Linear Unit (LeakyRELU), as a nonlinear activation function and a 3×3 kernel Conv filter layer [33], is part of the denoising encoder. This filter is a small matrix applied to the input data to extract local features by performing element-wise multiplications and summations. It captures spatial relationships between neighboring elements, balancing detailed feature extraction with the preservation of spatial resolution. This filter size is commonly used to maintain fine-grained details and enhance the accuracy of the reconstructed output. Furthermore, each layer undergoes Batch Normalization (BN), a technique that standardizes the inputs to a layer by recentering and rescaling them, which helps stabilize and accelerate the training process. Additionally, the feature compression of the denoising encoder transforms the channel matrix into a reshaped vector. This step is followed by a split of the channel matrix into two separate flows, namely a Fully Connected Network (FCN) and the DeCNN. The FCN performs as an integrated network that works on the acceleration of convergence and the mitigation of the vanishing gradient problem [34]. DeCNN utilizes a denoising module that effectively eliminates noise in the noisy channel matrix [19].

The DeCNN block has two layers: input and output, along with three denoising blocks. The three denoising blocks are connected in sequence, which enhances the effectiveness of the denoising process. Each of the three denoising blocks consists of a residual subnetwork with $N_{D}\;$layers and an element-wise subtraction operation. The first $N_{D}-1$ layer of the residual subnetwork utilizes the “Conv+BN+ReLU” operation. To analyze the spatial characteristics of the channel vector, the two operations of Conv and ReLU are used together. To ensure that network stability is enhanced and that network training is accelerated, BN is integrated between the two operations. A Conv operation is employed in the final layer of the residual subnetwork. This operation merges the extracted features and generates the residual noise vector, which is then used in subsequent element-wise subtraction. To utilize the additive character of noise, an element-wise subtraction is finally used to denoise the noisy channel vector, then the two vectors are added to generate the final codeword. The codeword is subsequently delivered to the BS via the feedback link for CSI recovery.

In BS, the reconstructed channel coefficient matrix, $\hat{H}$, is recovered through the use of the received codeword. The denoising decoder involves feature decompression and channel recovery. The feature decompression module is designed to include two flows, which are realized with the compressed method developed in the denoised encoder by an FCN and DeCNN block. The two flows are combined to produce the reconstructed output: $N_{c}\times N_{B}\times 2$ matrices, which estimate the real and imaginary components of $\hat{H}$ as an initial point. The channel recovery module reconstructs the channel matrix using two RefineNets units, which instantaneously refine reconstruction. The RefineNet unit consists of two layers: one input and one output, along with three further Conv layers, which utilize 3×3 kernels. This can be seen in Figure 3. The subsequent steps involve passing the refined channel matrix into the final Conv and BN layer. This is where the sigmoid function is employed to adjust the values within the range of [0,1] and produce the final reconstructed channel matrix $\hat{H},$ with the dimension $N_{c}\times N_{B}\times 2$.

The CsiNet-DeCNN model, introduced in [19] and illustrated briefly in this subsection, significantly enhanced the performance of the CsiNet in reconstructing the channel matrix, with a low NMSE and high channel estimation accuracy. However, it did not consider the time correlation in time-varying channels.

We improved the performance of this model by leveraging the DO technique and GRU unit, which will be detailed in the following subsection.

### 4.2. Gated Recurrent Unit (GRU)

**Figure 4:**
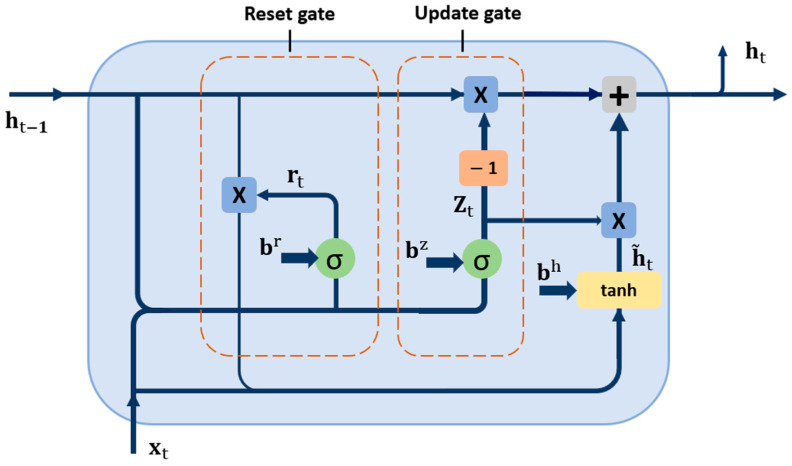
GRU and its architecture.

To enhance the performance of the system, GRU was proposed. The GRU [35] accelerates the model and improves accuracy by simplifying the architecture of LSTM networks [36]. Both GRU and LSTM use gating mechanisms to regulate the flow of information. In LSTM, three gates, such as the forget gate, input gate, and output gate manage data flow to and from the cell state, supporting long-term dependency management. On the other hand, the GRU streamlines the same process with only two gates: the update gate, which combines the function of the forget and input gates, and the reset gate, as shown in Figure 4.

To control the amount of information saved between the different states (current and previous), the update gate, $z_{t}$, is used, which is defined as [35]

(10) $$z_{t}=\sigma \;(W^{z}x_{t}+U^{z}h_{t-1}+b^{z})$$

The input weight matrix for $z_{t}$ is denoted by $W^{z}$. $b^{z}$ represents the bias term corresponding to this matrix, $h_{t-1}\;$represents the previous hidden state at time t−1, $U^{z}$ denotes the recurrent weight matrix, $x_{t}$ represents the GRU unit’s input vector, and σ represents the activation function for the two gates, update, and reset. The latter, $r_{t}$, responsible for specifying the amount of information about the previous moment, which can be defined as [35]

(11) $$r_{t}=\sigma \;(W^{r}x_{t}+U^{r}h_{t-1}+b^{r})$$

where $W^{r}$ is the input weight matrix for the reset gate, $b^{r}$ represents the corresponding bias term and $U^{r}$ denotes the recurrent weight matrix for the reset gate. Both candidate and output hidden state gates ${\tilde{h}}_{t}$ and $h_{t}$, respectively, are represented by [35]

(12) $${\tilde{h}}_{t}=tanh\;(W^{h}x_{t}+U^{h}(r_{t}\;{}^{\circ}\;h_{t-1})+b^{h})$$

(13) $$h_{t}=\left(1-z_{t}\right)\;{}^{\circ}\;h_{t-1}+z_{t}\;{}^{\circ}\;h_{t}$$

where $W^{h}$ represents the weight matrix of the output hidden state, while the corresponding bias term is $b^{h}$ and $U^{h}$ is the recurrent weight matrix. tanh represents the activation functions for the candidate gate.

To enhance the robustness and generalization capability of the proposed model, a DO technique [17] is applied to the final reconstructed channel matrix $\hat{H}$ from the CsiNet-DeCNN decoder. This is denoted by:

(14) $$H_{DO}=Dropout(\hat{H},p)$$

where p is the dropout rate. Subsequently, the processed sequence $H_{DO}$ is fed into the GRU unit. The GRU architecture allows for capturing temporal dependencies within the decoded feature across time steps t. At each time step t, the GRU computes the hidden state $h_{t}$ based on the input $H_{DO}(t)$ and the previous hidden state $h_{t-1}$, for t time steps, as follows:

(15) $$h_{t}=GRU(H_{DO}(t),\;h_{t-1})$$

### 4.3. The Proposed DGD-CNet Model Architecture

**Figure 5:**
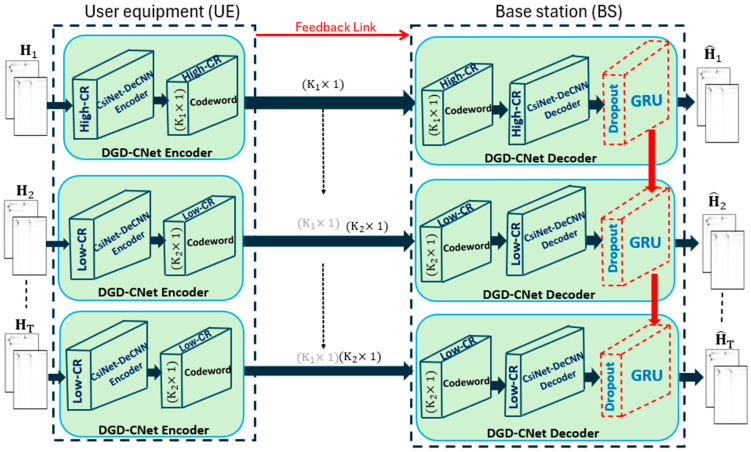
The proposed DGD-CNet model.

Inspired by RNN’s superior performance in channel spatial–temporal feature extraction, our proposed model improves CsiNet-DeCNN by leveraging DO and GRU to enhance the trade-off between CR and recovery quality. Figure 5 shows the proposed DGD-CNet model.

The two stages that comprise our model are the extraction of features from the angular-delay domain and the representation and final reconstruction of correlations. For feature extraction in the angular-delay domain, the model compresses CsiNet-DeCNN with two distinct CRs to ${\left\{H_{t}\right\}}_{t=1}^{T}$, to understand the structure of the angular-delay domain. The first channel, $H_{1}$, gets converted with the usage of CsiNet-DeCNN, with a High-Compression Ratio (High-CR). The conversion turns it into a codeword vector, ($K_{1}\times 1$), which can be used for high-resolution recovery, since it keeps enough structured information.

At the Low-Compression Ratio (Low-CR), the CsiNet-DeCNN encoder is used to process the remaining channel matrices of (T−1). This leads to the production of a sequence of ($K_{2}\times 1$) codewords ($K_{1}$ > $K_{2}$), since channel correlation reduces the amount of information needed. (T−1) codewords and the initial $\left(K_{1}\times 1\right)$ codeword are joined, before input into the Low-CR CsiNet-DeCNN decoder.

This ensures that feedback information is fully used. As features are extracted from the angular-delay domain, each CsiNet-DeCNN decoder produces two matrices, with sizes $N_{c}\times N_{B}$.

Since all the Low-CR CsiNets-DeCNNs in Figure 5 have the same job, they have identical weights and biases. This means that their network parameters are identical. With the constant change in speed and frequency of feedback, the value of T also changes, and an easy rescale of the architecture is possible. This ensures the performance of channel groups with a changing T. This condition decreases the parameter overhead. Rather than producing (T−1) copies, a Low-CR CsiNet-DeCNN is reapplied (T−1) times in operation.

To enhance the generalization and mitigate overfitting, the reconstructed channel matrix from the CsiNet-DeCNN decoder incorporates the DO technique, as described in Equation (14). Also, the proposed model is improved by integrating the GRU units. To be more specific, GRU receives lengthy T sequences as input from the DO as in Equation (15). During each step, previous inputs work on implicitly training the GRU, so that it gets to know time correlation. This is followed by their merger with current inputs to increase the recovery quality Low-CR.

The hidden layers in the GRU are equal to the output dimension. For the final reconstructed channel matrix, ${\hat{H}}_{t}$, two matrices made of $N_{c}\times N_{B}$ are reshaped from the final outputs. This matrix is then transformed into the spatial frequency domain using 2D-IDFT to obtain the CSI representation.

The procedure of the proposed DGD-CNet model is summarized as follows. At the UE, there are several CRs, CsiNet-DeCNN encoders are established, whereas at the BS, there are CsiNet-DeCNN decoders, as well as the DO-GRU system. Each side has a counter. $H_{1}$ is first compressed at the UE with High-CR, and it is subsequently recovered at the BS by a High-CR CsiNet-DeCNN decoder and DO-GRU. For t (2≤t≤T), marking the following time step, at the UE, $s_{t}$ is the result of the transformation of $H_{t}$ to a lower-dimensional codeword. The latter, $s_{t}$, should hold the learned correlation information. At the BS, $c_{t}$, which marks the lower dimensional codeword, is received. A concatenation takes place between $c_{t}$ and $c_{1}$ (received from $H_{1}$). Then, they are inversely transformed using CsiNet-DeCNN decoder and DO-GRU, at the BS. The counter makes an additional count following each step. The same operation takes place until T is reached on the counter. Then, a reset of the GRU takes place to be able to recover subsequent channel groups.

The overall network can be defined as an autoencoder. For this, it is assumed that fully differentiable channel models are used for training. This takes places for all kernels and biases for encoders and decoders, denoted by θ$=\{\theta_{enc}$,$\theta_{dec}\}$. The function of this autoencoder is denoted by f(.), which takes the input $H_{t}$ and produces the reconstructed channel matrix ${\hat{H}}_{t}$:

(16) $${\hat{H}}_{t}=f\left(H_{t};\theta \right)=f_{dec}(f_{enc}\left(H_{t};\theta_{enc}\right);\theta_{dec})$$

where θ represents the complete set of encoder–decoder parameters, f (·) represents the function of the network, and ${\hat{H}}_{t}$ is the reconstructed channel coefficient matrix of the cascaded CSI. For fair comparison with the other models in the literature, end-to-end training is accomplished on the network. For this to happen, a parameter update is undertaken by the Adaptive Moment Estimation Algorithm (ADAM) to minimize the Mean Squared Error (MSE) [37]. The loss function is defined as the MSE between the reconstructed channel ${\hat{H}}_{t}$ and the original channel $H_{t}$. It is calculated with the usage of the following Equation [26]:

(17) $$Loss=\frac{1}{N}\sum_{n=1}^{N}\sum_{t=1}^{T}{\left\|{\hat{H}}_{n,t}-H_{n,t}\right\|}_{F}^{2}$$

where N is the total number of samples in the training set.

### 4.4. Key Performance Indexes

To evaluate the performance of the proposed DGD-CNet model in enhancing channel estimation, several key performance indexes are utilized. The NMSE measures the variation between $\hat{H}$ and **H** (the reconstructed and the original channel matrix, respectively), and can be calculated from the Equation below [26]:

(18) $$NMSE=E\left\{\frac{1}{T}\sum_{t=1}^{T}\frac{{\left\|H_{t}-{\hat{H}}_{t}\right\|}_{F}^{2}}{{\left\|H_{t}\right\|}_{F}^{2}}\right\}$$

The correlation coefficient (ρ) measures the similarity between the original channel vector $h_{n,t}$ and the reconstructed channel vector ${\hat{h}}_{n,t}$ of the n˗th sub-carrier. This is carried out for a specific time t. Equation (19) is used to calculate (ρ) [26]:

(19) $$\rho =E\left\{\frac{1}{T}\frac{1}{{\tilde{N}}_{c}}\sum_{t=1}^{T}\sum_{n=1}^{{\tilde{N}}_{c}}\frac{\left|{\hat{h}}_{n,t}^{H}h_{n,t}\right|}{{\left\|{\hat{h}}_{n,t}\right\|}_{2}{\left\|h_{n,t}\right\|}_{2}}\right\}$$

where ${\hat{h}}_{n,t}\;$is the n˗th sub-carrier’s reconstructed channel vector at time t. The ratio of the reconstructed to original channel vector indicates accuracy, which can be described using the following Equation [38]:

(20) $$Accuracy=E\left\{\frac{1}{T}\frac{1}{{\tilde{N}}_{c}}\sum_{t=1}^{T}\sum_{n=1}^{{\tilde{N}}_{c}}\frac{\left|{\hat{h}}_{n,t}^{H}\right|}{{\left\|h_{n,t}\right\|}_{2}}\right\}$$

**Figure 6:**
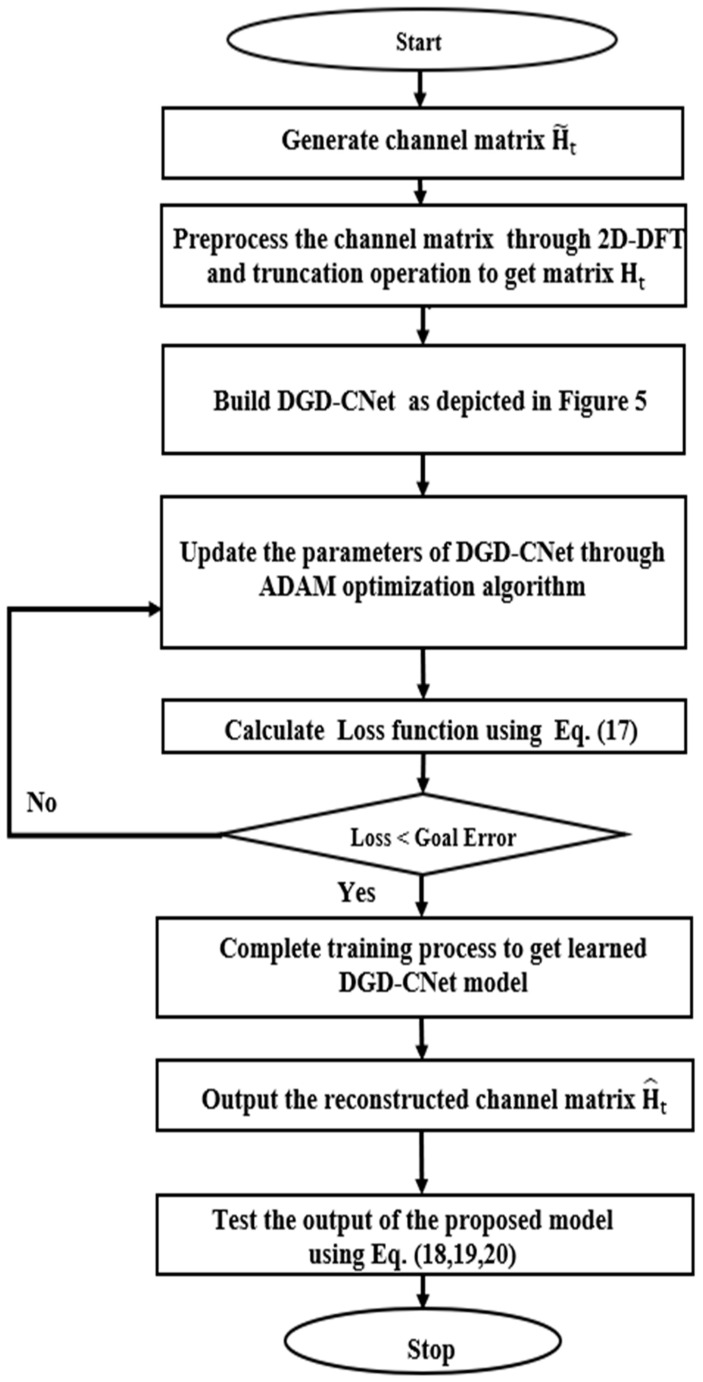
Flow chart of the training and testing process for the proposed DGD-CNet model.

These key performance indexes collectively provide a comprehensive assessment of the proposed DGD-CNet model’s effectiveness in various scenarios and conditions. The overall training and testing process for the proposed model can be summarized in the flowchart seen in Figure 6.

## 5. Numerical Results

This section provides the validation and analysis of the proposed DGD-CNet model and its comparison with the other models listed in the literature. Two types of channel matrices were generated using the channel model created by COST 2100 [39] to create the training and testing samples. Switching to the angular-delay domain and following truncation, the channel matrix H, i.e., was changed from size 1024×32 to size 32×32. Table 2 lists the channel setup parameters that apply to both indoor and outdoor situations, as well as the number of samples employed in testing, validation, and training to ensure fair comparison with the other existing models in the literature. These parameters include the number of epochs, batch size, and learning rate.

Collaboratory (Python 3.7) is used to implement the proposed DGD-CNet model. The performance of the proposed model is compared with other DL-based models that use CsiNet as their baseline. CsiNet offers a robust framework for channel estimation due to its advanced DL architecture, which enhances the channel estimation accuracy.

As such, the comparison undertaken in this paper with other models includes CsiNet [24], CsiNet-LSTM [26], Conv-LSTMCsiNet [27], CsiNet-DeCNN [19], and ACNet [30]. Using different CRs, NMSE is one of the parameters tested, along with the correlation coefficient (ρ), SNR, and accuracy in both indoor and outdoor situations.

For various CRs, the DGD-CNet model’s performance is evaluated. $H_{1}$ is compressed under 1/4 CR for all evaluations.

**Figure 7:**
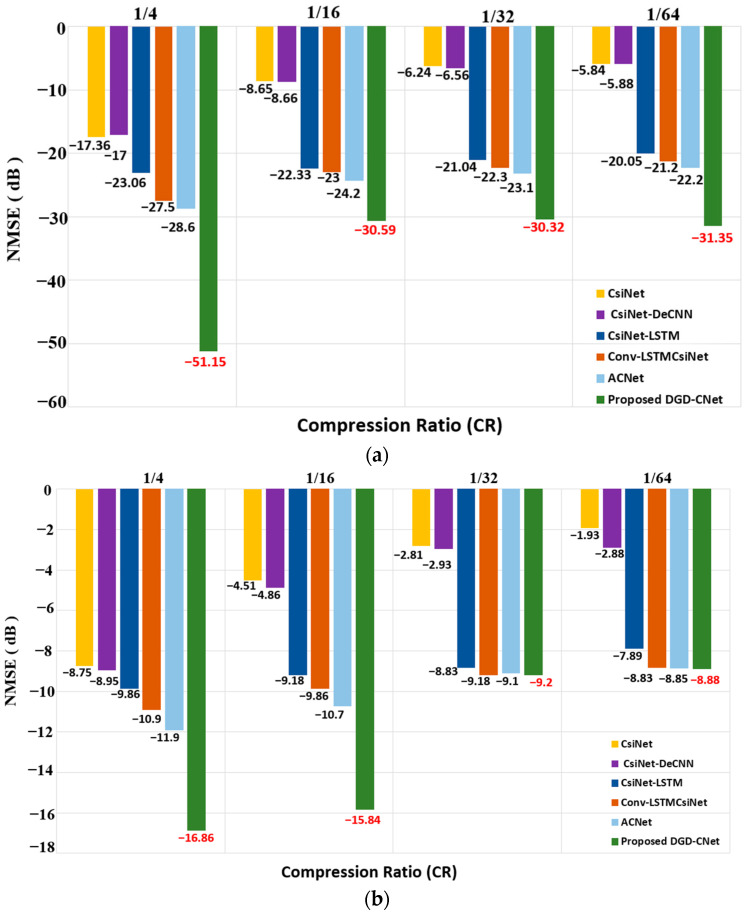
NMSE (dB) comparison of the proposed DGD-CNet model and other DL-based models at different CRs: (**a**) indoor and (**b**) outdoor at SNR = 5 dB. The red color represents the lowest NMSE value.

Figure 7 shows the comparison between NMSE (in dB) and different CRs for the proposed DGD-CNet model and other DL-based models in both indoor and outdoor situations at SNR 5 dB. This investigation used an SNR value of 5 dB to reflect a realistic and challenging scenario for channel estimation and compression ratio trade-offs.

For High-CR, the proposed model has better performance than other DL-based models, with the lowest NMSE at −51.15 dB and −16.86 dB for indoor and outdoor situations, respectively. When compared to previous DL-based models, the proposed model had the lowest performance loss for both situations at Low-CR.

To clarify the conducted comparison, Table 3 shows the percentage improvement in NMSE of the proposed model for Low-CR in comparison with other models from the literature (DL-based models). The model discussed in this paper shows that its performance improvement increases as CR decreases (Low-CR) for indoor situations. Also, for outdoor situations, our proposed model still shows better performance when CR decreases. For indoor situations, the improvements reach up to 437% over CsiNet and 433% over CsiNet-DeCNN at CR 1/64. In outdoor situations, the model shows up to 360% better NMSE compared to CsiNet at CR 1/64, although the gains are relatively lower due to the more complex and dynamic nature of outdoor environments. Nonetheless, the proposed model still outperforms other DL-based models, demonstrating its robustness and effectiveness in compressing CSI without compromising accuracy, making it a promising solution for CSI feedback in FDD-based IRS-aided Massive MIMO systems.

**Figure 8:**
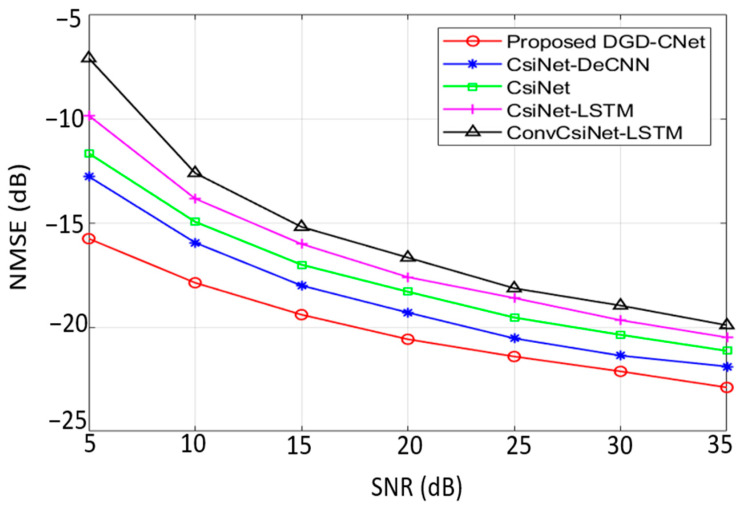
NMSE (dB) comparison of the proposed DGD-CNet model and other DL-based models at different SNR for outdoor situations.

Figure 8 provides a comparison between NMSE performance versus SNR, measured in dB, for different SNRs in outdoor situations at a 1/16 CR, the proposed DGD-CNet model consistently performs better than any other model across different SNR levels. This is an indication of the extent of its performance stability. Results also show that the DGD-CNet could adapt to a variety of noise levels and is resilient in different communication scenarios.

For an effective comparison between the DGD-CNet model and other DL-based models, CsiNet is employed as a baseline. For every tested scenario, the proposed model performed better than CsiNet. This same result was reached when compared with CsiNet-DeCNN, which includes an IRS component. This marks a validation of the design that includes both DO and GRU. Additionally, DGD-CNet surpasses in channel reconstruction over both CsiNet-LSTM and ConvCsiNet-LSTM, with GRU playing a significant role in this improvement.

Regarding indoor and outdoor situations, Table 4 shows the relationship linking CR to the correlation coefficient (ρ). The analysis suggests that the proposed DGD-CNet model achieves high-quality compression, evidenced by its ability to maintain high correlation coefficients in both indoor and outdoor situations.

At a higher CR of 1/4, DGD-CNet establishes a strong baseline with *ρ* values of 0.99 for indoor and 0.90 for outdoor situations. As the CR decreases to 1/16, 1/32, and 1/64, representing an increasingly higher level of data compression, DGD-CNet consistently demonstrates improvements over other models. Compared to its counterparts, DGD-CNet shows notable enhancement in both indoor and outdoor situations: at 1/16 CR, it achieves a 2% improvement indoors and 1% outdoors, maintaining robust performance across varying levels of data retention. Even at the lowest CR of 1/64, DGD-CNet exhibits a 2% enhancement indoors and 1% outdoors, highlighting its resilience in preserving channel information integrity under more severe data compression conditions. These findings underscore DGD-CNet’s effectiveness and suitability for enhancing channel estimation accuracy in FDD-based IRS-aided Massive MIMO systems, promising advancements in practical wireless communication applications.

Table 5 illustrates the relationship between different CRs and the accuracy of the other DL-based models for all situations, indoor and outdoor. The DGD-CNet model proposed here outperforms other models seen in previously published works in both situations because it has higher values of accuracy for different CRs. At a CR of 1/4, DGD-CNet achieves the highest accuracy, with 0.91 for indoor (an improvement of 10% over the next best model) and 0.75 for outdoor situations (an improvement of 4% over the next best model). As the CR decreases to 1/16, 1/32, and 1/64, indicating higher compression and lower data retention, the accuracy of all models declines. However, DGD-CNet consistently maintains superior performance, showing notable resilience. At a CR of 1/16, DGD-CNet achieves an accuracy of 0.65 indoors (an improvement of 4% over the next best model) and 0.53 outdoors (an improvement of 1% over the next best model), outperforming its counterparts. Even at the lowest CR of 1/64, DGD-CNet achieves higher accuracy (0.61 indoors and 0.40 outdoors) than other models, which struggle to maintain comparable performance. These results highlight the robustness and effectiveness of the proposed DGD-CNet in high-compression scenarios, making it a promising solution for improving channel estimation accuracy in FDD-based IRS-aided Massive MIMO systems.

The superior performance of DGD-CNet can be attributed to the integration of GRU and DO techniques within the CsiNet-DeCNN framework. The ability of the GRU to capture temporal dependencies in channel data, coupled with its simpler architecture, results in a more accurate CSI estimation. Additionally, the inclusion of DO plays a critical role in preventing overfitting and enhancing the generalization capabilities of the model. These elements work together to deliver significant improvements in NMSE and correlation coefficient, ultimately leading to a more accurate and robust performance compared to other DL-based models in the literature.

Table 6 displays the reconstructed images of the channel matrix for the proposed DGD-CNet model and the other models in the literature, compared with the original pseudo-gray image at different CRs. The images clearly illustrate the superior performance of the DGD-CNet model across all compression levels. At a higher CR of 1/4, all models produce reconstructions that closely resemble the original image, but the DGD-CNet retains slightly more detail. As the CR decreases to 1/16, the difference becomes more pronounced, with DGD-CNet maintaining more of the original features and providing a clearer image than CsiNet, CsiNet-LSTM, and ConvLSTM-CsiNet. This trend continues at a CR of 1/32, where the DGD-CNet’s reconstruction still holds more structural details compared to the more blurred and degraded image produced by other models.

At the lowest CR of 1/64, the DGD-CNet significantly outperforms all the other models, preserving more of the essential features and minimizing information loss, whereas all the other model’s reconstruction shows considerable degradation and loss of critical details. These visual differences underscore the effectiveness of the proposed DGD-CNet model in retaining higher fidelity to the original image, especially at lower CRs.

Table 7 compares the proposed DGD-CNet model and other models in the literature, specifically CsiNet and ConvLSTM-CsiNet, in terms of the number of parameters and floating-point operations per second (FLOPs). The number of parameters refers to the number of learnable weights within a model. This directly affects the model size, memory requirements, and the potential for overfitting. FLOPs represent the total number of operations the model performs during computation. It provides insight into the computational resources required to run the model.

The proposed DGD-CNet model presents notable advantages regarding the number of model parameters. Compared to CsiNet and ConvLSTM-CsiNet, which have significantly more parameters, the proposed model demonstrates a substantial reduction in the number of parameters. This is due to its streamlined model design, the use of GRUs, and the integration of DeCNN for effective feature processing. Additionally, incorporating DO helps prevent overfitting and enhances the model generalization performance.

However, this improvement in the number of parameters comes with a trade-off: the model exhibits more FLOPs than ConvLSTM-CsiNet. FLOPs are increased by the complex operations performed by GRUs and the DeCNN component. While the proposed model design minimizes the number of parameters and benefits from DO regularization effect, the higher FLOPs highlight the need to balance parameter efficiency with computational complexity.

Additionally, the computational complexity (FLOPs) of the proposed model decreases as the CR decreases (less data information), while aligning with the model’s objective to efficiently manage reduced data information. Specifically, the proposed model achieves a 71.41% reduction in complexity compared to CsiNet at High-CR, and an impressive 95.5% reduction in complexity at low-CR.

## 6. Conclusions

This paper proposes a new model named DGD-CNet for channel estimation to address feedback compression in FDD-based IRS-aided Massive MIMO systems. The DGD-CNet model was evaluated alongside various DL-based models, including CsiNet, Conv-LSTMCsiNet, CsiNet-LSTM, CsiNet-DeCNN, and ACNet. The evaluation focused on the NMSE, correlation coefficient, system accuracy, SNR, and computational complexity.

Compared to the conventional CsiNet, the DGD-CNet achieves a 437% improvement in NMSE at Low-CR for indoor scenarios and an 8% enhancement in the correlation coefficient over the CsiNet-DeCNN model. This significant improvement of the NMSE is primarily attributed to the integration of GRU and DO within the CsiNet-DeCNN framework. The GRU, with its simpler architecture, effectively captures temporal dependencies in the channel data while mitigating the risk of overfitting. This results in an improved generalization and more accurate CSI estimation. On the other hand, DO helps prevent overfitting by randomly setting a fraction of the units to zero during training. This prevents the model from becoming too dependent on any single neuron, thereby enhancing its robustness, accuracy, and contribution to a reduction in NMSE.

Despite its advantages, including a low NMSE, high accuracy, high correlation coefficient, and lower number of parameters, the DGD-CNet model has higher FLOPs compared to the ConvLSTM-CsiNet model; thus as a future work, it is required to study how to reduce those FLOPs for the DGD-CNet model.

The proposed model is particularly well-suited for 5G and future 6G networks, where precise channel estimation is crucial for optimizing performance and managing complex environments with high user densities. Future efforts may also involve collaborating with implementation teams involved in the manufacturing of reflecting surfaces to obtain experimental results.

## Figures and Tables

**Table 1:** Comparison of previous work with the proposed model.

Reference	Duplexing Mode	IRS Usage	Link Type	Channel Estimation Model
[22]	FDD	No	Feedback link	Compressive sensing
[23]			Feedback link	Compressive sensing
[24]			Feedback link	CsiNet
[26]			Feedback link	CsiNet-LSTM
[27]			Feedback link	Conv-LSTMCsiNet
[28]			Uplink and downlink	ADNN and ALSTM
[29]			Feedback link	IdasNet
[11]	TDD	Yes	Uplink	IDRSN
[12]			Uplink	ResU-Net
[13]			Uplink	DL-based CNN
[14]			Uplink	CDRN
[19]	FDD	Yes	Feedback link	CsiNet-DeCNN
[30]			Feedback link	ACNet
Proposed model			Feedback link	DGD-CNet

**Table 2:** Parameters set for the simulation process.

Parameter	Value	
MIMO OFDM bandwidth	20 MHz	
Channel matrix (H)	32 × 32	
$\mathrm{Number}\;\mathrm{of}\;\mathrm{base}\;\mathrm{station}\;\mathrm{antennas}\;(N_{B})$	32	
Number of IRS elements (M)	32	
$\mathrm{Number}\;\mathrm{of}\;\mathrm{sub}-\mathrm{carriers}\;({\tilde{N}}_{c})$	1024	
Number of training samples	100,000	
Number of validation samples	60,000	
Number of testing samples	20,000	
Number of epochs	1000	
Learning rate	0.001	
Batch size	200	
Dropout rate (p)	0.1	
Channel group size (T)	10	
$\mathrm{Feedback}\;\mathrm{time}\;(\delta_{t})$ (in seconds)	0.04	
Compression Ratio (CR)	1/4, 1/16, 1/32, 1/64	
**Channel model**	**Indoor Situation** [39]	**Outdoor Situation** [39]
Carrier frequency	5.3 GHz	300 MHZ
Velocity (in km/h)	0.0036	4.24
Coherent time (T_c_) (in seconds)	30	0.56

**Table 3:** NMSE improvement for the proposed DGD-CNet model compared to other DL-based models at Low-CR.

NMSE Improvement (%)							
		Indoor			Outdoor		
	CR	1/16	1/32	1/64	1/16	1/32	1/64
Compared Model							
**CsiNet**		254%	386%	437%	251%	227%	360%
**CsiNet-DeCNN**		253%	362%	433%	226%	214%	208%
**Conv-LSTMCsiNet**		33%	36%	48%	61%	0.2%	0.6%
**CsiNet-LSTM**		37%	44%	56%	73%	4%	12.5%
**ACNet**		26%	31%	41%	48%	1%	0.3%

**Table 4:** Comparison between the proposed DGD-CNet model and other DL-based models in terms of the correlation coefficient (ρ) at different CRs in indoor and outdoor situations.

CR	Model	Correlation Coefficient (ρ) Epoch = 1000	
		Indoor	Outdoor
**1/4**	CsiNet [24]	0.98	0.87
	CsiNet-LSTM [26]	**0.99**	**0.91**
	Conv-LSTMCsiNet [27]	0.95	0.90
	CsiNet-DeCNN [19]	**0.99**	0.88
	ACNet [30]	0.98	0.90
	Proposed DGD-CNet	**0.99**	0.90
**1/16**	CsiNet [24]	0.90	0.79
	CsiNet-LSTM [26]	0.94	0.79
	Conv-LSTMCsiNet [27]	0.93	0.78
	CsiNet-DeCNN [19]	0.91	0.79
	ACNet [30]	0.94	0.79
	Proposed DGD-CNet	**0.96**	**0.80**
**1/32**	CsiNet [24]	0.83	0.67
	CsiNet-LSTM [26]	0.86	0.68
	Conv-LSTMCsiNet [27]	0.85	0.68
	CsiNet-DeCNN [19]	0.86	0.68
	ACNet [30]	0.89	0.69
	Proposed DGD-CNet	**0.90**	**0.76**
**1/64**	CsiNet [24]	0.83	0.60
	CsiNet-LSTM [26]	0.80	0.60
	Conv-LSTMCsiNet [27]	0.84	0.62
	CsiNet-DeCNN [19]	0.84	0.69
	ACNet [30]	0.90	0.69
	Proposed DGD-CNet	**0.92**	**0.71**

The bold numbers represent the highest ρ value

**Table 5:** Accuracy comparison of the proposed DGD-CNet model and other DL-based models at different CRs in indoor and outdoor situations.

CR	Model	Accuracy Epoch = 1000	
		Indoor	Outdoor
**1/4**	CsiNet [24]	0.81	0.68
	CsiNet-LSTM [26]	0.80	0.67
	Conv-LSTMCsiNet [27]	0.81	0.70
	CsiNet-DeCNN [19]	0.82	0.70
	ACNet [30]	0.85	0.71
	Proposed DGD-CNet	**0.91**	**0.75**
**1/16**	CsiNet [24]	0.60	0.49
	CsiNet-LSTM [26]	0.59	0.49
	Conv-LSTMCsiNet [27]	0.60	0.49
	CsiNet-DeCNN [19]	0.61	**0.53**
	ACNet [30]	0.62	0.52
	Proposed DGD-CNet	**0.65**	**0.53**
**1/32**	CsiNet [24]	**0.51**	0.36
	CsiNet-LSTM [26]	0.48	0.35
	Conv-LSTMCsiNet [27]	**0.51**	0.36
	CsiNet-DeCNN [19]	0.50	**0.42**
	ACNet [30]	**0.51**	0.40
	Proposed DGD-CNet	**0.51**	**0.42**
**1/64**	CsiNet [24]	0.48	0.26
	CsiNet-LSTM [26]	0.52	0.20
	Conv-LSTMCsiNet [27]	0.53	0.26
	CsiNet-DeCNN [19]	0.50	0.28
	ACNet [30]	0.54	0.39
	Proposed DGD-CNet	**0.61**	**0.40**

The bold numbers represent the highest accuracy.

**Table 6:** The reconstruction image of the proposed DGD-CNet model and other models in the literature in comparison to the original pseudo-gray image.

CR	Original	CsiNet	CsiNet-LSTM	ConvLSTM-CsiNet	Proposed DGD-CNet
**1/4**	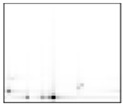	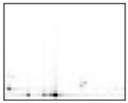 ρ=0.98	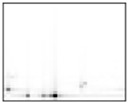 ρ=0.99	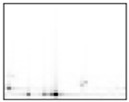 ρ=0.95	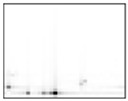 ρ=0.99
**1/16**	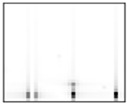	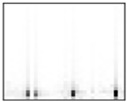 ρ=0.90	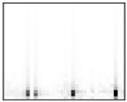 ρ=0.94	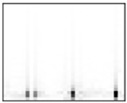 ρ=0.93	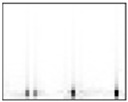 ρ=0.96
**1/32**	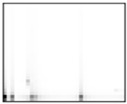	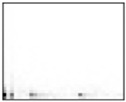 ρ=0.83	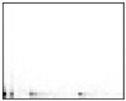 ρ=0.86	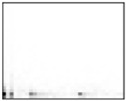 ρ=0.85	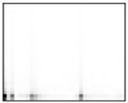 ρ=0.90
**1/64**	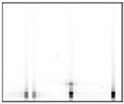	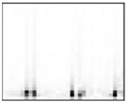 ρ=0.83	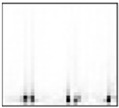 ρ=0.80	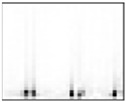 ρ=0.84	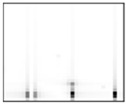 ρ=0.92

**Table 7:** Comparison of the number of parameters and FLOPs for the proposed model compared to the other models in the literature.

	CR	CsiNet	ConvLSTM-CsiNet	Proposed DGD-CNet
**Number of parameters**	1/4	2.1 M	2.1 M	**245.7 K**
	1/16	1.0 M	542.9 K	**61.4 K**
	1/32	530.6 K	280.7 K	**15.3 K**
	1/64	268.4 K	149.6 K	**3.8 K**
**FLOPs**	1/4	412.9 M	**44.5 M**	294.9 M
	1/16	409.8 M	**41.3 M**	117.9 M
	1/32	409.2 M	**40.8 M**	73.7 M
	1/64	409.0 M	40.5 M	**18.4 M**

The bold numbers represent the lowest number of parameters and FLOPs.

## Data Availability

Data are contained within the article.
